# Hesitant Fuzzy Soft Sets with Application in Multicriteria Group Decision Making Problems

**DOI:** 10.1155/2015/806983

**Published:** 2015-01-20

**Authors:** Jian-qiang Wang, Xin-E Li, Xiao-hong Chen

**Affiliations:** School of Business, Central South University, Changsha 410083, China

## Abstract

Soft sets have been regarded as a useful mathematical tool to deal with uncertainty. In recent years, many scholars have shown an intense interest in soft sets and extended standard soft sets to intuitionistic fuzzy soft sets, interval-valued fuzzy soft sets, and generalized fuzzy soft sets. In this paper, hesitant fuzzy soft sets are defined by combining fuzzy soft sets with hesitant fuzzy sets. And some operations on hesitant fuzzy soft sets based on Archimedean t-norm and Archimedean t-conorm are defined. Besides, four aggregation operations, such as the HFSWA, HFSWG, GHFSWA, and GHFSWG operators, are given. Based on these operators, a multicriteria group decision making approach with hesitant fuzzy soft sets is also proposed. To demonstrate its accuracy and applicability, this approach is finally employed to calculate a numerical example.

## 1. Introduction

Since the fuzzy set (FS) was proposed by Zadeh in 1965 [1], it has been widely studied, developed, and successfully applied in various fields, such as multicriteria decision making (MCDM) [2, 3], fuzzy logic and approximate reasoning [4], and pattern recognition [5]. In real MCDM cases, due to the fuzziness and uncertainty of decision making problems, the criteria's weights and evaluation values of alternatives can be inaccurate, uncertain, or incomplete. For the problems like those, FSs, especially fuzzy numbers, can provide good solutions. However, in FSs the membership degree of the element is represented by a single value between zero and one, and a major drawback of FSs is that single values cannot convey information precisely.

In practice, the information regarding alternatives, when referring to a fuzzy concept, may be incomplete; that is, the sum of the membership and nonmembership degree of an element in the universe can be less than one. The FS fails when it comes to managing the insufficient understanding of membership degrees. Thus, Atanassov's intuitionistic fuzzy sets (IFSs), interval-valued intuitionistic fuzzy sets (IVIFSs), and trapezoidal or triangular intuitionistic fuzzy sets, as the extensions of Zadeh's FSs, were introduced [6–11]. IFSs and IVIFSs have been widely applied in solving MCDM problems [10, 12–14].

However, in some cases, the membership degree of an element is neither a single value nor an interval, but a set of possible values. To manage such situations where decision-makers are hesitant in expressing their preferences over alternatives, hesitant fuzzy sets (HFSs), another extension of traditional FSs, provide a useful reference. HFSs are first introduced by Torra [15, 16] and permit the membership degree of an element to be a set of several possible values between 0 and 1. HFSs are tremendously useful in handling the situations where people have hesitancy in providing their preferences over objects in a decision making process. The aggregation operators of HFSs were studied and applied to MCDM problems in [17–20]. Besides, Wang et al. [21] provided an outranking approach with HFSs to solve MCDM problems. Yu et al. [22] and Chen et al. [23] discussed the correlation coefficients of HFSs and their applications to clustering analysis. Xu and Xia [24, 25] discussed the distance and correlation measures for HFSs.

However, in some practical cases, FSs and their extensions failed to model uncertain data being of various types because of their inadequacy as a parameterization tool. To overcome this difficulty, Molodtsov [26] proposed soft sets (SSs), which were considered as a useful mathematical tool for dealing with uncertainties which is free from the difficulties affecting the exiting methods. After that, many scholars have shown an intense interest in this. Maji et al. [27] gave a theoretical study of SSs. They [28] also described the application of SSs in a decision making problem. In addition, the operations of SSs were extended in [29–31]. Min [32] proposed the similarity measures between SSs. Aktaş and Çağman [33] gave a definition of soft groups and discussed the basic properties. Acar et al. [34] introduced soft rings. Gong et al. [35] proposed bijective SSs and the corresponding operations. Furthermore, the relations and functions of SSs were studied by Babitha and Sunil [36]. Ali et al. studied the algebraic structures of SSs [37] and also discussed the idea of reduction of parameters in case of SSs [38]. Çağman and Enginoğlu [39] defined the products of SSs and uni-int decision function and then constructed a uni-int decision making method. Feng et al. [40] improved Çağman and Enginoğlu's uni-int decision making method based on choice value being in the form of SSs.

But situations in real world may be complex because of the fuzzy nature of parameters. To deal with such situation, Maji et al. [41] extended classical SSs to fuzzy soft sets (FSSs). Borah et al. [42] discussed some operations of FSSs. Guan et al. [43] gave a new order relation of FSSs and Feng et al. [44] studied the decomposition of FSSs with finite value spaces. Similar to FS theory, several new extensional concepts based on FSSs are given. For example, Maji et al. [45] introduced intuitionistic fuzzy soft sets (IFSSs) by integrating SSs with IFSs. To overcome the difficulties of representation of parameter's vagueness, Xu et al. [46] introduced vague soft set (VSSs) and Zhou and Li [47] defined generalized vague soft sets (GVSSs). By combining IVFSs and SSs, Yang et al. [48] proposed interval-valued fuzzy soft sets (IVFSSs). Ma et al. [49] analyzed the parameter reduction of IVFSSs and Jiang et al. [50] studied the entropy of IFSSs and IVFSSs. In addition, Majumdar and Samanta [51] defined generalized fuzzy soft sets (GFSSs). Moreover, FSSs have been also successfully applied in MCDM problems in recent years. Roy and Maji [52] gave an approach to decision making problems. By means of level soft sets, Feng et al. [53] presented an adjustable approach to FSSs based decision making. Kong et al. [54] presented a decision making algorithm of FSSs based on grey theory. Mitra Basu et al. [55] proposed a balanced solution of FSSs in medical science. Xiao et al. [56] integrated the fuzzy cognitive map and FSSs for solving the supplier selection problem. In [57, 58], the approaches to MCDM based on IFSSs are given. Jiang et al. [59] presented an adjustable approach to IFSSs-based decision making by using level soft sets of IFSSs. To deal with the problems of subjective evaluation and uncertain knowledge, Xiao et al. [60] proposed an evaluation method based on GFSSs and its application in medical diagnosis problem. They also [61] extended classical SSs to trapezoidal fuzzy soft sets (TFSSs) and applied them to MCDM problems. Zhang et al. [62] applied generalized TFSSs to medical diagnosis. Zhang [63] presented a rough set approach to IFSSs-based decision making.

IFSSs [45], VSSs [46], IVFSSs [48], and GFSSs [51] are all proposed to deal with uncertainties by taking advantages of SSs. In those extensions of SSs, the value of membership is either a single value or an interval. But in fact, the membership degree may be a set of possible values in a SS, so the purpose of this paper is to deal with this situation by combining HFSs with FSSs. To do this, a new kind of SSs, hesitant fuzzy soft sets (HFSSs), can be defined. HFSSs can represent various different preferences from different decision-makers and avoid overlooking any subjective intentions of decision-makers. Babitha and John [64] introduced HFSSs and analyzed some basis operations. However, the MCDM method proposed by Babitha and John [64] was not persuadable. In fact, HFSSs are more suitable for multiple criteria group decision making (MCGDM) problems. In this paper, a further study of HFSSs and their application in MCGDM problems are given.

The rest of this paper is organized as follows. In Section 2, some basic concepts of t-norm, t-conorm, SSs, FSSs, and HFSs are briefly reviewed. In Section 3, the concept of HFSSs and the corresponding operations are introduced. In Section 4, some aggregation operators of HFSSs are given. Based on hesitant fuzzy soft numbers (HFSNs), a MCGDM approach is proposed in Section 5. An illustrative example is given in Section 6 and the conclusions are provided in Section 7.

## 2. Preliminaries

In this section, some basic concepts of t-norm, t-conorm, SSs, FSSs, and HFSs are reviewed.

### 2.1. T-Norm and T-Conorm

**Definition 1 (see [65, 66]).**

A function *T* : [0,1]×[0,1]→[0,1] is called a t-norm if the following conditions are true:

1. for  all  *x* ∈ [0,1], *T*(1, *x*) = *x*;
2. for  all  *x*, *y* ∈ [0,1], *T*(*x*, *y*) = *T*(*y*, *x*);
3. for  all  *x*, *y*, *z* ∈ [0,1], *T*(*x*, *T*(*y*, *z*)) = *T*(*T*(*x*, *y*), *z*);
4. if *x* ≤ *x*′, *y* ≤ *y*′, then *T*(*x*, *y*) = *T*(*x*′, *y*′).

**Definition 2 (see [65, 66]).**

A function *S* : [0,1]×[0,1]→[0,1] is called a t-conorm if the following four conditions are true:

1. for  all  *x* ∈ [0,1], *S*(0, *x*) = *x*;
2. for  all  *x*, *y* ∈ [0,1], *S*(*x*, *y*) = *S*(*y*, *x*);
3. for  all  *x*, *y*, *z* ∈ [0,1], *S*(*x*, *S*(*y*, *z*)) = *S*(*S*(*x*, *y*), *z*);
4. if *x* ≤ *x*′, *y* ≤ *y*′, then *S*(*x*, *y*) = *S*(*x*′, *y*′).

**Definition 3 (see [65, 66]).**

A t-norm function *T*(*x*, *y*) is called Archimedean t-norm if it is continuous and, for  all  *x* ∈ (0,1), *T*(*x*, *x*) < *x*. If for  all  *x*, *y* ∈ (0,1), *T*(*x*, *y*) is strictly increasing, *T*(*x*, *y*) can be called strictly Archimedean t-norm. A t-conorm function *S*(*x*, *y*) is called Archimedean t-conorm if it is continuous and, for  all  *x* ∈ (0,1), *S*(*x*, *x*) < *x*. If for  all  *x*, *y* ∈ (0,1), *S*(*x*, *y*) is strictly increasing, *S*(*x*, *y*) can be called strictly Archimedean t-conorm.

It is well known [67] that a strictly Archimedean t-norm is generated by its additive generator *k* as *T*(*x*, *y*) = *k* ^−1^(*k*(*x*) + *k*(*y*)) and *k* is a strictly decreasing function: [0,1]→[0, *∞*) such that *k*(1) = 0. Let *l*(*t*) = *k*(1 − *t*), and then Archimedean t-conorm can be expressed as *S*(*x*, *y*) = *l* ^−1^(*l*(*x*) + *l*(*y*)).

If we use specific forms to represent *k*, then some t-norms and t-conorms can be obtained.

1. Assuming *k*(*t*) = −log⁡*t*, then *l*(*t*) = −log⁡(1 − *t*), *k* ^−1^(*t*) = *e* ^−*t*^, and *l* ^−1^(*t*) = 1 − *e* ^−*t*^. Algebraic t-conorm and t-norm [68] can be obtained: *S* ^*A*^(*x*, *y*) = *x* + *y* − *xy*, and *T* ^*A*^(*x*, *y*) = *xy*.
2. Assuming *k*(*t*) = log⁡((2 − *t*)/*t*), then *l*(*t*) = log⁡((2 − (1 − *t*))/(1 − *t*)), *k* ^−1^(*t*) = 2/(*e* ^*t*^ + 1), and *l* ^−1^(*t*) = 1 − (2/(*e* ^*t*^ + 1)). Einstein t-conorm and t-norm [68] can be obtained: *S* ^*E*^(*x*, *y*) = (*x* + *y*)/(1 + *xy*), and *T* ^*E*^(*x*, *y*) = *xy*/(1 + (1 − *x*)(1 − *y*)).

### 2.2. Soft Sets and Fuzzy Soft Sets

In this subsection, the definitions of SSs and FSSs are introduced.

**Definition 4 (see [26]).**

Let *U* be an initial universe and let *E* be a set of parameters. A pair (*F*, *E*) is called soft set (SS) over *U*, where *F* is a mapping of *E* into the set of all subsets of *U*.

In other words, any SS is a parameterized family of subsets of the set *U*. For  all  *e* ∈ *E*, *F*(*e*) may be considered the set of elements of the sets (*F*, *E*), or the set of *e*-approximate elements of the SS. To illustrate this idea, let us consider the following example.

**Example 5 .**

Suppose that *U* = {*h*_1_, *h*_2_, *h*_3_, *h*_4_} is a set of houses and *E* = {*e*_1_, *e*_2_, *e*_3_} is a set of parameters, which stand for being beautiful, being cheap and being in the green surroundings, respectively. Consider the mapping from parameter set *E* to the set of all subsets of *U*. Then SS (*F*, *E*) can describe an “attractive” house that Mr. X is going to buy:

(1) $$\begin{array}{c} F\left(e_{1}\right)=\left\{h_{2},h_{4}\right\},\;\;F\left(e_{2}\right)=\left\{h_{1},h_{3}\right\},\\ F\left(e_{3}\right)=\left\{h_{3},h_{4}\right\}. \end{array}$$

Thus, we can view the SS (*F*, *E*) as a collection of approximations as follows: (*F*, *E*) = {beautiful houses = {*h*_2_, *h*_4_}, cheap houses = {*h*_1_, *h*_3_}, in the green surroundings houses = {*h*_3_, *h*_4_}}.

For the purpose of storing a SS in a computer, we could represent the SS of Example 5 in Table 1.

**Definition 6 (see [41]).**

Let $\tilde{F}(U)$ be the set of all fuzzy subsets of *U*, and then a pair $\left(\tilde{F},E\right)$ is called a fuzzy soft set (FSS) over $\tilde{F}(U)$, where $\tilde{F}$ is a mapping denoted by

(2) $$\begin{array}{c} \tilde{F}:E⟶\tilde{F}\left(U\right). \end{array}$$

**Example 7 .**

Consider Example 5. If Mr. X thinks *h*_1_ is a little expensive and this fuzzy information cannot be expressed only by two crisp numbers, that is, 0 and 1, a membership degree can be used instead, which is associated with each element and represented by a real number in the interval [0,1]. Then FSS $\left(\tilde{F},E\right)$ can describe the “attractive” house that Mr. X is going to buy under the fuzzy information:

(3) $$\begin{array}{c} \tilde{F}\left(e_{1}\right)=\left\{\frac{h_{1}}{0.2},\frac{h_{2}}{0.7},\frac{h_{3}}{0.1},\frac{h_{4}}{0.7}\right\},\\ \tilde{F}\left(e_{2}\right)=\left\{\frac{h_{1}}{0.8},\frac{h_{2}}{0.3},\frac{h_{3}}{0.7},\frac{h_{4}}{0.1}\right\},\\ \tilde{F}\left(e_{3}\right)=\left\{\frac{h_{1}}{0.1},\frac{h_{2}}{0.2},\frac{h_{3}}{0.9},\frac{h_{4}}{0.8}\right\}. \end{array}$$

Similarly, the representation of the FSS of Example 7 can be shown in Table 2.

### 2.3. Hesitant Fuzzy Sets

**Definition 8 (see [15]).**

Let *X* be a universal set, and then a hesitant fuzzy set (HFS) on *X* is defined in terms of a function *h* that when applied to *X* returns a finite subset of [0,1]. A HFS can be represented by

(4) $$\begin{array}{c} E=\left\{\langle x,h_{E}\left(x\right)\rangle \;|\;x\in X\right\}, \end{array}$$

where *h*_*E*_(*x*) is a set of values in [0, 1], denoting the possible membership degrees of the element *x* ∈ *X* to the set* E*. *h*_*E*_(*x*) is called a hesitant fuzzy element (HFE) [17], and *H* is the set of all HFEs. In particular, if *X* has only one element, we call *E* a hesitant fuzzy number (HFN), briefly denoted by *E* = {*h*_*E*_(*x*)}. The set of all hesitate fuzzy numbers is represented as HFNs.

Torra [15] defined some operations on HFNs, and Xia and Xu [17] defined some new operations on HFNs and also the score functions.

**Definition 9 (see [15]).**

Let *h*_1_, *h*_2_, *h* ∈ *H*, and three operations are defined as follows:

1. *h* ^*c*^ = ∪_*γ*∈*h*_{1 − *γ*};
2. *h*_1_ ∪ *h*_2_ = ∪_*γ*_1_∈*h*_1_,*γ*_2_∈*h*_2__max⁡{*γ*_1_, *γ*_2_};
3. *h*_1_∩*h*_2_ = ∩_*γ*_1_∈*h*_1_,*γ*_2_∈*h*_2__min⁡{*γ*_1_, *γ*_2_}.

The arithmetical operations of HFNs based on Archimedean t-norm and Archimedean t-conorm are defined as follows.

**Definition 10 (see [17]).**

Let *h*_1_ = ∪_*γ*_1_∈*h*_1__{*γ*_1_}, *h*_2_ = ∪_*γ*_2_∈*h*_2__{*γ*_2_}, and *h* = ∪_*γ*∈*h*_{*γ*} be three HFNs, and *λ* ≥ 0. Four operations are defined as follows:

1. *h* ^*λ*^ = ∪_*γ*∈*h*_{*k* ^−1^(*λk*(*γ*))};
2. *λh* = ∪_*γ*∈*h*_{*l* ^−1^(*λl*(*γ*))};
3. *h*_1_ ⊕ *h*_2_ = ∪_*γ*_1_∈*h*_1_,*γ*_2_∈*h*_2__{*l* ^−1^(*l*(*γ*_1_) + *l*(*γ*_2_))};
4. *h*_1_ ⊗ *h*_2_ = ∪_*γ*_1_∈*h*_1_,*γ*_2_∈*h*_2__{*k* ^−1^(*k*(*γ*_1_) + *k*(*γ*_2_))}.
5. In particular, if *k*(*t*) = −log⁡*t*, then
6. *h* ^*λ*^ = ∪_*γ*∈*h*_{*γ* ^*λ*^};
7. *λh* = ∪_*γ*∈*h*_{1 − (1 − *γ*)^*λ*^};
8. *h*_1_ ⊕ *h*_2_ = ∪_*γ*_1_∈*h*_1_,*γ*_2_∈*h*_2__{*γ*_1_ + *γ*_2_ − *γ*_1_ *γ*_2_};
9. *h*_1_ ⊗ *h*_2_ = ∪_*γ*_1_∈*h*_1_,*γ*_2_∈*h*_2__{*γ*_1_ *γ*_2_}.
10. If *k*(*t*) = log⁡((2 − *t*)/*t*), then
11. *h* ^*λ*^ = ∪_*γ*∈*h*_{2*γ* ^*λ*^/((2 − *γ*)^*λ*^ + *γ* ^*λ*^)};
12. *λh* = ∪_*γ*∈*h*_{((1 + *γ*)^*λ*^ − (1 − *γ*)^*λ*^)/((1 + *γ*)^*λ*^ + (1 − *γ*)^*λ*^)};
13. *h*_1_ ⊕ *h*_2_ = ∪_*γ*_1_∈*h*_1_,*γ*_2_∈*h*_2__{(*γ*_1_ + *γ*_2_)/(1 + *γ*_1_ *γ*_2_)};
14. *h*_1_ ⊗ *h*_2_ = ∪_*γ*_1_∈*h*_1_,*γ*_2_∈*h*_2__{*γ*_1_ *γ*_2_/(1 + (1 − *γ*_1_)(1 − *γ*_2_))}.

**Definition 11 (see [17]).**

For *h* ∈ HFN, *s*(*h*) = (1/#*h*)∑_*γ*∈*h*_ *γ* is called the score function of *h*, where #*h* is the number of elements in *h*. For two HFNs and *h*_2_, if *s*(*h*_1_) > *s*(*h*_2_), then *h*_1_ > *h*_2_; if *s*(*h*_1_) = *s*(*h*_2_), then *h*_1_ = *h*_2_.

It is clear that Definition 11 does not consider the situation where two HFNs *h*_1_ and *h*_2_ have the same score, but their deviation degrees may be different. The deviation degree of all elements with respect to the average value in a HFN reflects how elements are consistent with each other, that is, whether they have a higher consistency or not. To better represent this issue, Chen et al. [69] defined the deviation degree as follows.

**Definition 12 (see [69]).**

For *h* ∈ HFN, *σ*(*h*) = [(1/#*h*)∑_*γ*∈*h*_(*γ*−*s*(*h*))^2^]^1/2^ is defined as the variance of *h*, where *s*(*h*) is the score function of *h*, and *σ*(*h*) denotes the deviation degree of *h*.

**Definition 13 (see [69]).**

Let *h*_1_ and *h*_2_ be two HFNs; if *s*(*h*_1_) > *s*(*h*_2_), then *h*_1_ > *h*_2_.

If *s*(*h*_1_) = *s*(*h*_2_), then

1. if *σ*(*h*_1_) > *σ*(*h*_2_), then *h*_1_ < *h*_2_;
2. if *σ*(*h*_1_) < *σ*(*h*_2_), then *h*_1_ > *h*_2_;
3. if *σ*(*h*_1_) = *σ*(*h*_2_), then *h*_1_ = *h*_2_.

## 3. Hesitant Fuzzy Soft Sets and Their Operations

In this section, a HFSS is defined by combining HFSs and SSs; some operations on HFSSs based on Archimedean t-norm and Archimedean t-conorm are also given.

### 3.1. Definition of Hesitant Fuzzy Soft Sets

HFSs and SSs are integrated as mentioned in Section 2. The concept of HFSSs is defined as follows.

**Definition 14 .**

Let *U* be an universe, let *E* be a set of parameters, and let $\tilde{F}(U)$ be the set of all hesitant fuzzy subsets of *U*. A pair $\left(\tilde{F},E\right)$ is called a HFSS over *U*, where $\tilde{F}$ is a mapping denoted by

(5) $$\begin{array}{c} \tilde{F}:E⟶\tilde{F}\left(U\right). \end{array}$$

A HFSS is a parameterized family of hesitant fuzzy subsets of *U*, that is, $\tilde{F}(U)$. For all $\varepsilon \in E,\tilde{F}(\varepsilon )$ is referred to as the set of *ε*-approximate elements of the HFSS $\left(\tilde{F},E\right)$. It can be written as

(6) $$\begin{array}{c} \tilde{F}\left(\varepsilon \right)=\left\{\langle x,\mu_{\tilde{F}\left(\varepsilon \right)\left(x\right)}\rangle \;|\;x\in U\right\}. \end{array}$$

Since HFE can represent the situation, in which different membership functions are considered possible [15], $\mu_{\tilde{F}(\varepsilon )(x)}$ is a set of several possible values, which is the hesitant fuzzy membership degree. In particular, if $\tilde{F}(\varepsilon )$ has only one element, $\tilde{F}(\varepsilon )$ can be called a hesitant fuzzy soft number (HSSN). For convenience, a HSSN is denoted by $\tilde{F}(\varepsilon )=\left\{\langle x,\mu_{\tilde{F}\left(\varepsilon \right)\left(x\right)}\rangle \right\}$.

**Example 15 .**

Consider Example 7. Mr. X found it was hard to give a single value to express his opinion about the houses with respect to different criteria. For example, Mr. X thinks that the degree of house *h*_1_ satisfies that criterion *e*_1_ “beautiful” is 0.3 or 0.2. Then a HFSS $\left(\tilde{F},E\right)$ can be used to describe the “attractive” house that Mr. X is going to buy:

(7) F~e1=h10.3,0.2,h20.8,0.7,h30.2,0.1,h40.7;F~e2=h10.9,0.8,h20.3,h30.7,0.6,h40.1;F~e3=h10.1,h20.3,0.2,h30.9,0.7,h40.8.

For the purpose of storing a HFSS in a computer, the HFSS of Example 15 is shown in Table 3.

### 3.2. Operations on Hesitant Fuzzy Soft Sets

In this subsection, some operations on HFSNs are defined.

**Definition 16 .**

Let *F*(*e*_*i*_) = {*h*_*p*_/*μ*_*ip*_∣*p* = 1,2,…, *m*} and *F*(*e*_*j*_) = {*h*_*p*_/*μ*_*jp*_∣*p* = 1,2,…, *m*} be two HFSNs and *λ* > 0. Then the following can be defined:

1. (*F*(*e*_*i*_))^*c*^ = {*h*_*p*_/∪_*γ*_*ip*_∈*μ*_*ip*__{1 − *γ*_*ip*_}∣*p* = 1,2,…, *m*};
2. *λF*(*e*_*i*_) = {*h*_*p*_/∪_*γ*_*ip*_∈*μ*_*ip*__{*l* ^−1^(*λl*(*γ*_*ip*_))}∣*p* = 1,2,…, *m*};
3. *F*(*e*_*i*_)^*λ*^ = {*h*_*p*_/∪_*γ*_*ip*_∈*μ*_*ip*__{*k* ^−1^(*kl*(*γ*_*ip*_))}∣*p* = 1,2,…, *m*};
4. *F*(*e*_*i*_) ⊕ *F*(*e*_*j*_) = {*h*_*p*_/∪_*γ*_*ip*_∈*μ*_*ip*_,*γ*_*jp*_∈*μ*_*jp*__{*l* ^−1^(*l*(*γ*_*ip*_) + *l*(*γ*_*jp*_))}∣*p* = 1,2,…, *m*};
5. *F*(*e*_*i*_) ⊗ *F*(*e*_*j*_) = {*h*_*p*_/∪_*γ*_*ip*_∈*μ*_*ip*_,*γ*_*jp*_∈*μ*_*jp*__{*k* ^−1^(*k*(*γ*_*ip*_) + *k*(*γ*_*jp*_))}∣*p* = 1,2,…, *m*}.

**Theorem 17 .**

Let *F*(*e*_*i*_) = {*h*_*p*_/*μ*_*ip*_∣*p* = 1,2,…, *m*} and *F*(*e*_*j*_) = {*h*_*p*_/*μ*_*jp*_∣*p* = 1,2,…, *m*} be two HFSNs and *λ*, *λ*_1_, *λ*_2_ > 0. Then the following are true:

1. *F*(*e*_*i*_) ⊕ *F*(*e*_*j*_) = *F*(*e*_*j*_) ⊕ *F*(*e*_*i*_);
2. *F*(*e*_*i*_) ⊗ *F*(*e*_*j*_) = *F*(*e*_*j*_) ⊗ *F*(*e*_*i*_);
3. *λ*(*F*(*e*_*i*_) ⊕ *F*(*e*_*j*_)) = *λF*(*e*_*i*_) ⊕ *λF*(*e*_*j*_);
4. (*F*(*e*_*i*_) ⊗ *F*(*e*_*j*_))^*λ*^ = *F*(*e*_*i*_)^*λ*^ ⊗ *F*(*e*_*j*_)^*λ*^;
5. *λ*_1_ *F*(*e*_*i*_) ⊕ *λ*_2_ *F*(*e*_*i*_) = (*λ*_1_ + *λ*_2_)*F*(*e*_*i*_);
6. (*F*(*e*_*i*_))^*λ*_1_^ ⊗ (*F*(*e*_*i*_))^*λ*_2_^ = (*F*(*e*_*i*_))^*λ*_1_+*λ*_2_^.

**Proof**

In Definition 16, it is easy to prove that (1) and (2) hold, and thus let us prove (3). Consider

(8) λ(F(ei)⊕F(ej))=λhp⋃γip∈μip,γjp∈μjpl−1lγip+lγjp ∣ p∈P=hp⋃γip∈μip,γjp∈μjpl−1λll−1lγip+lγjp ∣   hp⋃γip∈μip,γjp∈μjpl−1λll−1lγip+lγjpp∈P=hp⋃γip∈μip,γjp∈μjpl−1λlγip+lγjp ∣ p∈P;λF(ei)⊕λF(ej)=hp⋃γip∈μipl−1λlγip ∣ p∈P ⊕hp⋃γjp∈μjpl−1λlγjp ∣ p∈P=hp⋃γip∈μip,γjp∈μjpl−1ll−1λlγip+ll−1λlγjp−1           ⋃γip∈μip,γjp∈μjpl−1ll−1λlγip+ ll−1λlγjp−1 ∣   ⋃γip∈μip,γjp∈μjpl−1ll−1λlγipp∈P=hp⋃γip∈μip,γjp∈μjpl−1λlγip+lγjp ∣ p∈P=λFei⊕Fej,

where *P* = {1,2,…, *m*}.

Similarly, (4)–(6) of Definition 16 can be proved.

## 4. Aggregation Operators of Hesitant Fuzzy Soft Sets

In this section, some aggregation operators are defined.

**Definition 18 .**

Let *F*(*e*_*i*_) = {*h*_*p*_/*μ*_*ip*_∣*p* = 1,2,…, *m*}  (*i* = 1,2,…, *n*) be the elements of the HFSS $\left(\tilde{F},E\right)$, and let *ω* = (*ω*_1_, *ω*_2_,…, *ω*_*n*_) be the weight vector of *F*(*e*_*i*_)  (*i* = 1,2,…, *n*), where *ω*_*i*_ indicates the importance degree of *F*(*e*_*i*_), satisfying *ω*_*i*_ ≥ 0  (*i* = 1,2,…, *n*) and ∑_*i*=1_^*n*^ *ω*_*i*_ = 1. Then the HFSWA operator can be called hesitant fuzzy soft weighted averaging operator and defined as

(9) $$\begin{array}{c} \text{HFSWA}\left(F\left(e_{1}\right),F\left(e_{2}\right),\ldots ,F\left(e_{n}\right)\right)=\;\overset{n}{\underset{i=1}{⨁}}\omega_{i}F\left(e_{i}\right). \end{array}$$

**Theorem 19 .**

Let *F*(*e*_*i*_) = {*h*_*p*_/*μ*_*ip*_∣*p* = 1,2,…, *m*}  (*i* = 1,2,…, *n*) be the elements of the HFSS $\left(\tilde{F},E\right)$, and let *ω* = (*ω*_1_, *ω*_2_,…, *ω*_*n*_) be the weight vector of *F*(*e*_*i*_)  (*i* = 1,2,…, *n*), where *ω*_*i*_ indicates the importance degree of *F*(*e*_*i*_), satisfying *ω*_*i*_ ≥ 0  (*i* = 1,2,…, *n*) and ∑_*i*=1_^*n*^ *ω*_*i*_ = 1. Then the aggregated value by using the HFSWA operator is still a HFSN, and

(10) HFSWAFe1,Fe2,…,Fen= ⨁i=1nωiFei=hp⋃γip∈μipl−1∑i=1nωilγip ∣ p=1,2,…,m.

**Proof**

Use the mathematical introduction of *n* to complete this proof.

For *n* = 2, we have

(11) HFSWA Fe1,Fe2= ⨁i=12ωiF(ei)=ω1F(e1)⊕ω2F(e2)=hp⋃γ1p∈μ1p,γ2p∈μ2pl−1ω1lγ1p+ω2lγ2p ∣   hp⋃γ1p∈μ1p,γ2p∈μ2pl−1ω1lγ1p+ω2lγ2pp=1,2,…,m.

Suppose (10) holds for *n* = *k*; that is,

(12) HFSWAFe1,Fe2,…,Fek= ⨁i=1kωiF(ei)=hp⋃γip∈μipl−1∑i=1kωilγip ∣ p=1,2,…,m,HFSWAFe1,Fe2,…,Fek,Fek+1= ⨁i=1kωiF(ei)⊕ωi+1F(ei+1)=hp⋃γip∈μipl−1∑i=1kωilγip ∣ p=1,2,…,m ⊕hp⋃γk+1p∈μk+1pl−1λlγk+1p ∣ p=1,2,…,m=hp⋃γip∈μipl−1ll−1∑i=1kωilγip        l−1ll−1∑i=1kωilγip+ll−1ωk+1lγk+1p−1 ∣   ⋃γip∈μipl−1ll−1∑i=1kωilγipp=1,2,…,m=hp⋃γip∈μipl−1∑i=1kωilγip+ωk+1lγk+1p ∣   hp⋃γip∈μipl−1∑i=1kωilγip+ωk+1lγk+1pp=1,2,…,m=hp⋃γip∈μipl−1∑i=1k+1ωilγip ∣ p=1,2,…,m.

Now, (10) holds for *n* = *k* + 1, and thus (10) holds for all *n*.

Subsequently, some desirable properties of the HFSWA operator are investigated.

**Property 1 .**

If all *F*(*e*_*i*_)  (*i* = 1,2,…, *n*) are equal, that is, *F*(*e*_*i*_) = *F*(*e*) = {*h*_*p*_/*μ*_*ip*_∣*p* = 1,2,…, *m*} for all *i*, then

(13) $$\begin{array}{c} \text{HFSWA}\left(F\left(e_{1}\right),F\left(e_{2}\right),\ldots ,F\left(e_{n}\right)\right)=F\left(e\right). \end{array}$$

**Proof**

Let *F*(*e*_*i*_) = *F*(*e*), and then

(14) HFSWAFe1,Fe2,…,Fen=HFSWA(F(e),F(e),…,F(e))= ⨁i=1nωiF(e)=hp⋃γip∈μipl−1∑i=1nωilγip ∣ p=1,2,…,m=hp⋃γip∈μipl−1lγip ∣ p=1,2,…,m=hpμip ∣ p=1,2,…,m i=1,2,…,n=Fe.

**Property 2 .**

Let *F*(*e*_*i*_) = {*h*_*p*_/*μ*_*ip*_∣*p* = 1,2,…, *m*}  (*i* = 1,2,…, *n*) be the elements of the HFSS $\left(\tilde{F},E\right)$, and let *ω* = (*ω*_1_, *ω*_2_,…, *ω*_*n*_) be the weight vector of *F*(*e*_*i*_)  (*i* = 1,2,…, *n*) such that *ω*_*i*_ ≥ 0  (*i* = 1,2,…, *n*) and ∑_*i*=1_^*n*^ *ω*_*i*_ = 1. If *F*(*ε*) = {*h*_*p*_/*μ*_*εp*_∣*p* = 1,2,…, *m*} is a hesitant fuzzy soft element (HFSE), then

(15) HFSWAFe1⊕Fε,Fe2⊕Fε,…,Fen⊕Fε=HFSWAFe1,Fe2,…,Fen⊕Fε.

**Proof**

Since *F*(*e*_*j*_) ⊕ *F*(*ε*) = {*h*_*p*_/∪_*γ*_*jp*_∈*μ*_*jp*_,*γ*_*εp*_∈*μ*_*εp*__{*l* ^−1^(*l*(*γ*_*jp*_) + *l*(*γ*_*εp*_))}∣*p* = 1,2,…, *m*}, then

(16) HFSWA(F(e1)⊕F(ε),F(e2)⊕F(ε),…,F(en)⊕F(ε))=hp⋃γip∈μip,γεp∈μεpl−1∑i=1nωill−1lγip+lγεp ∣   hp⋃γip∈μip,γεp∈μεpl−1∑i=1nωill−1lγip+lγεpp=1,2,…,m=hp⋃γip∈μip,γεp∈μεpl−1∑i=1nωilγip+lγεp ∣   hp⋃γip∈μip,γεp∈μεpl−1∑i=1nωilγip+lγεpp=1,2,…,m,HFSWAFe1,Fe2,…,Fen⊕Fε=hp⋃γip∈μipl−1∑i=1nωilγip ∣ p=1,2,…,m ⊕hpμεp ∣ p=1,2,…,m=hp⋃γip∈μip,γεp∈μεpl−1ll−1∑i=1nωilγip         l−1ll−1∑i=1nωilγip+lγεp−1 ∣ p=1,2,…,m=hp⋃γip∈μip,γεp∈μεpl−1∑i=1nωilγip+lγεp ∣   hp⋃γip∈μip,γεp∈μεpl−1∑i=1nωilγip+lγεpp=1,2,…,m.

Therefore, HFSWA (*F*(*e*_1_) ⊕ *F*(*ε*), *F*(*e*_2_) ⊕ *F*(*ε*),…, *F*(*e*_*n*_) ⊕ *F*(*ε*)) = HFSWA (*F*(*e*_1_), *F*(*e*_2_),…, *F*(*e*_*n*_))⊕*F*(*ε*).

**Property 3 .**

Let *F*(*e*_*i*_) = {*h*_*p*_/*μ*_*ip*_∣*p* = 1,2,…, *m*}  (*i* = 1,2,…, *n*) be the elements of the HFSS $\left(\tilde{F},E\right)$, and let *ω* = (*ω*_1_, *ω*_2_,…, *ω*_*n*_) be the weight vector of *F*(*e*_*i*_)  (*i* = 1,2,…, *n*) such that *ω*_*i*_ ≥ 0  (*i* = 1,2,…, *n*) and ∑_*i*=1_^*n*^ *ω*_*i*_ = 1. If *λ* > 0, then

(17) HFSWAλFe1,λFe2,…,λFen=λ HFSWAFe1,Fe2,…,Fen.

**Proof**

According to Definition 16, *λF*(*e*_*i*_) = {*h*_*p*_/∪_*γ*_*ip*_∈*μ*_*ip*__{*l* ^−1^(*λl*(*γ*_*ip*_))}∣*p* = 1,2,…, *m*}.

Then

(18) HFSWAFe1,Fe2,…,Fen=hp⋃γip∈μipl−1∑i=1nωill−1λlγip ∣   hp⋃γip∈μipl−1∑i=1nωill−1λlγipp=1,2,…,m=hp⋃γip∈μipl−1∑i=1nωiλlγip ∣ p=1,2,…,m,λHFSWAFe1,Fe2,…,Fen=hp⋃γip∈μipl−1λll−1∑i=1nωilγip ∣   hp⋃γip∈μipl−1∑i=1nωill−1λlγipp=1,2,…,m=hp⋃γip∈μipl−1λ∑i=1nωilγip ∣ p=1,2,…,m.

Therefore, the proof of Property 3 is completed.

According to Properties 2 and 3, Property 4 can be given.

**Property 4 .**

Let *F*(*e*_*i*_) = {*h*_*p*_/*μ*_*ip*_∣*p* = 1,2,…, *m*}  (*i* = 1,2,…, *n*) be all the elements of the HFSS $\left(\tilde{F},E\right)$, and let *ω* = (*ω*_1_, *ω*_2_,…, *ω*_*n*_) be the weight vector of *F*(*e*_*i*_), such that *ω*_*i*_ ≥ 0  (*i* = 1,2,…, *n*) and ∑_*i*=1_^*n*^ *ω*_*i*_ = 1. If *λ* > 0 and *F*(*ε*) = {*h*_*p*_/*μ*_*εp*_∣*p* = 1,2,…, *m*} is a HFSE, then

(19) HFSWAλFe1⊕Fε,λFe2⊕Fε,…,    λF(en)⊕F(ε)=λHFSWAFe1,Fe2,…,Fen⊕Fε.

**Property 5 .**

Let *F*(*e*_*i*_) = {*h*_*p*_/*μ*_*ip*_∣*p* = 1,2,…, *m*}  (*i* = 1,2,…, *n*) be the elements of the HFSS $\left(\tilde{F},E_{1}\right)$, let *F*(*ε*_*i*_) = {*h*_*p*_/*μ*_*ε*_*i*_*p*_∣*p* = 1,2,…, *m*}  (*i* = 1,2,…, *n*) be the elements of HFSS $\left(\tilde{F},E_{2}\right)$, and let *ω* = (*ω*_1_, *ω*_2_,…, *ω*_*n*_) be the weight vector of them, satisfying *ω*_*i*_ ≥ 0  (*i* = 1,2,…, *n*) and ∑_*i*=1_^*n*^ *ω*_*i*_ = 1. Then

(20) HFSWAFe1⊕Fε1,Fe2⊕Fε2,…,Fen⊕FεnhhhhhhhFen⊕Fεn=HFSWAFe1,Fe2,…,Fen ⊕HFSWAFε1,Fε2,…,Fεn.

**Proof**

According to Definition 16, *F*(*e*_*i*_) ⊕ *F*(*ε*_*i*_) = {*h*_*p*_/∪_*γ*_*ip*_∈*μ*_*ip*_,*γ*_*ε*_*i*_*p*_∈*μ*_*jp*__{*l* ^−1^(*l*(*γ*_*ip*_) + *l*(*γ*_*ε*_*i*_*p*_))}∣*p* = 1,2,…, *m*}.

Then

(21) HFSWAFe1⊕Fε1,Fe2⊕Fε2,…,Fen⊕Fεn=hp⋃γip∈μip,γεip∈μεipl−1∑i=1nωill−1lγip+lγεip ∣   hp⋃γip∈μip,γεip∈μεipl−1∑i=1nωill−1lγip+lγεipp=1,2,…,m=hp⋃γip∈μip,γεip∈μεipl−1∑i=1nωilγip+lγεip ∣   hp⋃γip∈μip,γεip∈μεipl−1∑i=1nωilγip+lγεipp=1,2,…,m,HFSWAFe1,Fe2,…,Fen ⊕HFSWAFε1,Fε2,…,Fεn=hp⋃γip∈μipl−1∑i=1nωilγip ∣ p=1,2,…,m ⊕hp⋃γεi∈μεipl−1∑i=1nωilγεip ∣ p=1,2,…,m=hp⋃γip∈μip,γεi∈μεipl−1ll−1∑i=1nωilγip         ×ll−1∑i=1nωilγip             + ll−1l−1∑i=1nωilγip              ×∑i=1nωilγεip−1 ∣   ⋃γip∈μip,γεi∈μεipl−1ll−1∑i=1nωilγipp=1,2,…,m=hp⋃γip∈μip,γεi∈μεipl−1∑i=1nωilγip+∑i=1nωilγεip ∣   hp⋃γip∈μip,γεi∈μεipl−1∑i=1nωilγip+∑i=1nωilγεipp=1,2,…,m=hp⋃γip∈μip,γεip∈μεipl−1∑i=1nωilγip+lγεip ∣   hp∪γip∈μip,γεip∈μεipl−1∑i=1nωilγip+lγεipp=1,2,…,m.

Thus, the proof of Property 5 is completed.

**Definition 20 .**

Let *F*(*e*_*i*_) = {*h*_*p*_/*μ*_*ip*_∣*p* = 1,2,…, *m*}  (*i* = 1,2,…, *n*) be the elements of the HFSS $\left(\tilde{F},E\right)$, and let *ω* = (*ω*_1_, *ω*_2_,…, *ω*_*n*_) be the weight vector of *F*(*e*_*i*_)  (*i* = 1,2,…, *n*), where *ω*_*i*_ indicates the importance degree of *F*(*e*_*i*_), satisfying *ω*_*i*_ ≥ 0  (*i* = 1,2,…, *n*) and ∑_*i*=1_^*n*^ *ω*_*i*_ = 1. Then the HFSWG operator can be called hesitant fuzzy soft weighted geometric operator and defined as

(22) $$\begin{array}{c} \text{HFSWG}\left(F\left(e_{1}\right),F\left(e_{2}\right),\ldots ,F\left(e_{n}\right)\right)=\;\overset{n}{\underset{i=1}{⨁}}F{\left(e_{i}\right)}^{\omega_{i}}. \end{array}$$

**Theorem 21 .**

Let *F*(*e*_*i*_) = {*h*_*j*_/*p*_*j*_∣*j* = 1,2,…, *m*}  (*i* = 1,2,…, *n*) be the elements of the HFSS $\left(\tilde{F},E\right)$, and let *ω* = (*ω*_1_, *ω*_2_,…, *ω*_*n*_) be the weight vector of *F*(*e*_*i*_)  (*i* = 1,2,…, *n*), where *ω*_*i*_ indicates the importance degree of *F*(*e*_*i*_), satisfying *ω*_*i*_ ≥ 0  (*i* = 1,2,…, *n*) and ∑_*i*=1_^*n*^ *ω*_*i*_ = 1. Then the aggregated value by using HFSWG operator is a HFSN, and

(23) HFSWGFe1,Fe2,…,Fen= ⨁i=1nFeiωi=hp⋃γip∈μipk−1∑i=1nωikγip ∣ p=1,2,…,m.

Similarly, it is clear that the HFSWG operator also satisfies the properties that HFSWA operator has, and the relative details are omitted here.

**Definition 22 .**

Let *F*(*e*_*i*_)  (*i* = 1,2,…, *n*) be the elements of the HFSS $\left(\tilde{F},E\right)$, and let *ω* = (*ω*_1_, *ω*_2_,…, *ω*_*n*_) be the weight vector of *F*(*e*_*i*_)  (*i* = 1,2,…, *n*), where *ω*_*i*_ indicates the importance degree of *F*(*e*_*i*_), satisfying *ω*_*i*_ ≥ 0  (*i* = 1,2,…, *n*) and ∑_*i*=1_^*n*^ *ω*_*i*_ = 1. Then the GHFSWA operator can be called the generalized hesitant fuzzy soft weighted averaging operator and defined as

(24) GHFSWAλFe1,Fe2,…,Fen=⨁i=1nωiFeiλ1/λ.

In particular, if *λ* = 1, then the GHFSWA operator is reduced to the HFSWA operator.

**Theorem 23 .**

Let *F*(*e*_*i*_)  (*i* = 1,2,…, *n*) be the elements of the HFSS $\left(\tilde{F},E\right)$, and let *ω* = (*ω*_1_, *ω*_2_,…, *ω*_*n*_) be the weight vector of *F*(*e*_*i*_)  (*i* = 1,2,…, *n*), where *ω*_*i*_ indicates the importance degree of *F*(*e*_*i*_), satisfying *ω*_*i*_ ≥ 0  (*i* = 1,2,…, *n*) and ∑_*i*=1_^*n*^ *ω*_*i*_ = 1. Then the aggregated value by using GHFSWA operator is a HFSN, and

(25) GHFSWAλFe1,Fe2,…,Fen=⨁i=1nωiFeiλ1/λ=hp⋃γip∈μipk−11/λkl−1∑i=1nωilk−1λkγip ∣ p=1,2,…,m.

**Definition 24 .**

Let *F*(*e*_*i*_)  (*i* = 1,2,…, *n*) be the elements of the HFSS $\left(\tilde{F},E\right)$, and let *ω* = (*ω*_1_, *ω*_2_,…, *ω*_*n*_) be the weight vector of *F*(*e*_*i*_)  (*i* = 1,2,…, *n*), where *ω*_*i*_ indicates the importance degree of *F*(*e*_*i*_), satisfying *ω*_*i*_ ≥ 0  (*i* = 1,2,…, *n*) and ∑_*i*=1_^*n*^ *ω*_*i*_ = 1. Then the GHFSWG operator can be called the generalized hesitant fuzzy soft weighted geometric operator and defined as

(26) GHFSWGλFe1,Fe2,…,Fen=1λ⨁i=1nλFeiωi.

In particular, if *λ* = 1, then the GHFSWG operator is reduced to the HFSWG operator.

**Theorem 25 .**

Let *F*(*e*_*i*_)  (*i* = 1,2,…, *n*) be the elements of the HFSS $\left(\tilde{F},E\right)$, and let *ω* = (*ω*_1_, *ω*_2_,…, *ω*_*n*_) be the weight vector of *F*(*e*_*i*_)  (*i* = 1,2,…, *n*), where *ω*_*i*_ indicates the importance degree of *F*(*e*_*i*_), satisfying *ω*_*i*_ ≥ 0  (*i* = 1,2,…, *n*) and ∑_*i*=1_^*n*^ *ω*_*i*_ = 1. Then the aggregated value by using GHFSWG operator is a HFSN, and

(27) GHFSWGλFe1,Fe2,…,Fen=1λ⨁i=1nλFeiωi=hp⋃γip∈μipl−11/λlk−1∑i=1nωikl−1λlγip ∣   hp⋃γip∈μipl−11/λlk−1∑i=1nωikl−1λlγipp=1,2,…,m.

Similarly, it is clear that the GHFSWA and GHFSWG operators possess the same properties that the HFSWA operator has.

**Example 26 .**

Suppose *F*(*e*_1_) = {*h*_1_/{0.3,0.5}, *h*_2_/{0.4,0.6}} and *F*(*e*_2_) = {*h*_1_/{0.4,0.5}, *h*_2_/{0.8,0.9}} are the elements of the HFSS $\left(\tilde{F},E\right)$, and *ω* = (0.3,0.7) is the weight vector of *F*(*e*_*i*_)  (*i* = 1,2), which indicate the corresponding importance degrees.

Using Theorems 19 and 21, if we assign *k*(*t*) = −log⁡(*t*), then

(28) HFSWAFe1,Fe2= ⨁i=12ωiF(ei) =hp⋃γip∈μip1−∏i=121−γipωi ∣ p=1,2 =h10.37,0.45,0.43,0.50,h20.72,0.83,0.75,0.85;HFSWGFe1,Fe2= ⨁i=12Feiωi =hp⋃γip∈μip∏i=12γipωi ∣ p=1,2 =h10.37,0.43,0.43,0.50,h20.65,0.71,0.73,0.80.

## 5. Multicriteria Group Decision Making Approach with HFSSs

Consider a MCGDM with hesitant fuzzy soft information, and assume there are* n *alternatives *U* = {*h*_1_, *h*_2_,…, *h*_*n*_} and* m* criteria *E* = {*e*_1_, *e*_2_,…, *e*_*m*_}, and the weight vector of criteria is *ω* = (*ω*_1_, *ω*_2_,…, *ω*_*m*_), where *ω*_*j*_ ≥ 0  (*j* = 1,2,…, *m*) and ∑_*j*=1_^*m*^ *ω*_*j*_ = 1. Suppose that there are *k* decision-makers *D* = {*d*_1_, *d*_2_,…, *d*_*k*_}, whose corresponding weight vector is *η* = (*η*_1_, *η*_2_,…, *η*_*k*_). Then a MCGDM problem can be concisely expressed by *k* HFSSs as follows:

(29) $$\begin{array}{c} F_{k}\left(e_{i}\right)=\left\{\frac{h_{j}}{\mu_{ij}}\;|\;j=1,2,\ldots ,n\right\}\;\left(i=1,2,\ldots ,m\right). \end{array}$$

To obtain the best alternative, the steps of the proposed MCGDM approach with HFSSs are given as follows.

**Step 1 (normalize the decision information).**

For benefit-type criteria, no further action need be taken. However, for cost-type criteria, the negation operator given in Definition 16 need be used. The normalized information can be denoted as follows:

(30) Fei=hjμij,j∈BThj∪γij∈μij1−γij,j∈CThhhhhhi(i=1,2,…,m;  j=1,2,…,n).

Here, *B*_*T*_ is the set of benefit-type criteria and *C*_*T*_ is the set of cost-type criteria.

**Step 2 (aggregate the HFSEs of each decision-maker).**

Calculate the comprehensive evaluation values of each alternative for each decision-maker by using the GHFSWA or GHFSWG operator:

(31) Fke=GHFSWAλFke1,Fke2,…,Fken=⨁i=1nωiFkeiλ1/λ=hj⋃γij∈μijk−11/λkl−1∑i=1nωilk−1λkγij ∣ j=1,2,…,m i=1,2,…,n,

or

(32) Fke′=GHFSWGλFke1,Fke2,…,Fken=1λ⨁i=1nλFkeiωi=hj⋃γij∈μijl−11/λlk−1∑i=1nωikl−1λlγij ∣ j=1,2,…,m i=1,2,…,n.

**Step 3 (aggregate the comprehensive HFSEs of all decision-makers).**

Calculate the overall values by using the HFSWA or HFSWG operator:

(33) Fe=HFSWAF1e,F2e,…,Fke= ⨁i=1kηiFi(e)=hj⋃γij∈μijl−1∑i=1kηilγij ∣ j=1,2,…,m,

or

(34) Fe=HFSWGF1e′,F2e′,…,Fke′= ⨁i=1kFke′ηi=hj⋃γij∈μijk−1∑i=1kηikγij ∣ j=1,2,…,m.

**Step 4 .**

Rank the HFNs *μ*_*i*_ (*i* = 1,2,…, *n*) of the alternative *h*_*i*_ (*i* = 1,2,…, *n*) by using the ranking method described in Definitions 11–13.

**Step 5 .**

Rank all the alternatives and select the best one(s) in accordance with the ranking of *μ*_*i*_ (*i* = 1,2,…, *n*).

## 6. Illustrative Example

In this section, we cite the commonly used example [27] and extend it to the hesitant fuzzy soft environment.

Mr. X's family wants to buy an attractive house considering three criteria: being beautiful (*e*_1_), being cheap (*e*_2_) and being in the green surroundings (*e*_3_), respectively. The weight of the criteria is *ω* = (0.3,0.3,0.4). The family can make their choice among four houses *U* = {*h*_1_, *h*_2_, *h*_3_, *h*_4_}. Mr. X, his wife, and his son have their own opinions about the given houses, whose corresponding weight vector is (0.5,0.3,0.2). They make their evaluations for four houses according to three criteria *E* = {*e*_1_, *e*_2_, *e*_3_} and the evaluation values are in the form of HFSSs, as shown in Tables 4, 5, and 6.

### 6.1. The Decision Making Procedure Based on HFSSs

To obtain the best alternative, let *λ* = 1, *k*(*t*) = −log⁡(*t*), and then the following steps are given.

**Step 1 (normalize the decision information).**

The criteria are of benefit-type, so no transformation is required.

**Step 2 (aggregate the HFSEs of each decision-maker).**

By using the GHFSWA operator, the comprehensive evaluation values of Mr. X are

(35) F1e=GHFSWAF1e1,F1e2,F1e3=h10.568,0.468,0.551,0.448,h20.519,0.493,0.457,0.427,  h3{0.741,0.597,0.717,0.561,0.731,0.574,0.707,0.545},  h4{0.645}.

The comprehensive evaluation values of Mrs. X are

(36) F2e=GHFSWAF2e1,F2e2,F2e3=h10.848,h20.703,0.650,h30.482,h40.735.

The comprehensive evaluation values of Mr. X's son are

(37) F3e=GHFSWAF3e1,F3e2,F3e3=h10.703,0.650,h20.711,h30.532,h40.748,0.734.

By using the GHFSWG operator, the comprehensive evaluation values of Mr. X are

(38) F1e′=GHFSWGF1e1,F1e2,F1e3=h1{0.269,0.259,0.238,0.230},h2{0.403,0.342,0.387,0.329},  h3{0.532,0.481,0.508,0.459,0.432,0.390,0.412,0.373},  h4{0.412}.

The comprehensive evaluation values of Mrs. X are

(39) F2e′=GHFSWGF2e1,F2e2,F2e3=h10.839,h20.668,0.633,h30.422,h40.660.

The comprehensive evaluation values of Mr. X's son are

(40) F3e′=GHFSWGF3e1,F3e2,F3e3=h10.668,0.633,h20.663,h30.367,h40.682,0.638.

**Step 3 (aggregate the comprehensive HFSEs of all decision-makers).**

By utilizing the HFSWA operator, the overall HFSS *F*(*e*) can be obtained as follows:

(41) Fe=HFSWAF1e,F2e,F3e=h1{0.707,0.697,0.675,0.664,0.701,0.691,0.669,0.658},  h2{0.624,0.605,0.614,0.595,0.600,0.580,0.590,0.569},  h3{0.641,0.552,0.625,0.533,0.634,0.540,0.618,0.524},  h4{0.696,0.693}.

By utilizing HFSWG operator, the overall HFSS *F*(*e*′) can be obtained as follows:

(42) Fe′=HFSWGF1e′,F2e′,F3e′=h1{0.454,0.449,0.445,0.441,0.427,0.422,0.420,0.415},  h2{0.518,0.510,0.477,0.470,0.508,0.500,0.468,0.461},  h3{0.461,0.438,0.450,0.428,0.415,0.395,0.405,0.386},  h4{0.525,0.518}.

**Step 4 .**

Rank the HFNs *μ*_*i*_ (*i* = 1,2,…, 4) of the alternative *h*_*i*_ (*i* = 1,2,…, 4) by using the ranking method described in Definitions 11–13.

The ranking results of *s*(*μ*_*i*_) (*i* = 1,2, 3,4) are shown in Table 7.

**Step 5 .**

Rank all the alternatives and select the best one(s) in accordance with the ranking of *s*(*μ*_*i*_) (*i* = 1,2,…, 4).

Obviously, the best alternative is *h*_4_, and the worst alternative is *h*_3_.

### 6.2. Sensitivity Analysis and Discussion

In this subsection, different t-norms as well as the parameter *λ* are adopted to calculate the same example given in Section 6.1.

**Case 1 .**

If Einstein t-norm and t-conorm are used to handle hesitant fuzzy soft information, then *k*(*t*) = log⁡((2 − *t*)/*t*) and the ranking results of *s*(*h*_*i*_) (*i* = 1,2, 3,4) are shown in Table 8.

Obviously, the rankings of the alternatives in Table 8, got by using *k*(*t*) = log⁡((2 − *t*)/*t*), are completely the same as those got by using *k*(*t*) = −log⁡(*t*), shown in Table 7. So using different t-norms could lead to no influence on the results.

**Case 2 .**

In the following, in order to illustrate the influence of the parameter *λ* on this example, we use different values of *λ* in Step 2 of the proposed approach to rank the alternatives. And the ranking results are shown in Tables 9 and 10.

From Tables 9 and 10, it can be seen that if the value of *λ* is one, then the best alternative is *h*_4_ and the worst alternative is *h*_3_, no matter which operators are used in Step 2 of the proposed approach. However, either the GHFSWA or GHFSWA operator is used in Step 2, and different *λ* for the same operator may lead to different aggregation results and the final ranking of alternatives is also different as the parameter changes. On the other hand, if *λ* remains the same, the results obtained by using the GHFSWA operator are also different from those by the GHFSWG operator. By using the GHFSWA operator, decision-makers emphasize the comprehensive priority of all criteria. By using the GHFSWG operator, decision-makers avoid choosing the alternatives that have poor performances in some criteria. Apparently, regardless of using different operators or different *λ*, the worst alternative is always *h*_3_ while the best alternative may be one of the other three alternatives. Thus, Mr. X's family can properly select the desirable alternative according to their interests and the actual needs.

In a word, the developed approach based on different t-norms and t-conorms can be used to deal with different relationships among the aggregated arguments. It can handle MCGDM problems in a flexible and objective manner under hesitant fuzzy soft environment and can provide more choices for decision-makers. At the same time, different results may be obtained by using different aggregation operators, which reflected the preferences of decision-makers. The main advantages of the approach proposed in this paper are not only its ability to effectively deal with the preference information expressed by HFSNs, but also its consideration that different t-norm and t-conorms can lead to different aggregation operators. This can avoid losing and distorting the preference information provided, which makes the final results suitably correspond with real life decision making problems.

## 7. Conclusion

In this paper, we introduced hesitant fuzzy soft sets, which can be regarded as an extension of both a SS and a HFS. HFSSs can describe the real preferences of decision-makers and reflect their uncertainty and hesitancy, which permit the membership degree to have a set of possible values in SSs. This paper also focuses on MCGDM problems in which the preferences of decision-makers are expressed by HFSNs. The new approach to solving these problems is based on the aggregation operators of HFSNs. Having reviewed the relevant literatures, operations of HFSNs based on Archimedean t-norm and Archimedean t-conorm are defined. Then four aggregation operators such as the HFSWA, HFSWG, GHFSWA, and GHFSWA operators are developed. Different t-norms and different parameters in generalized operators can be used in MCGDM methods. Finally, a numerical example is provided to illustrate the advantages of the proposed approach. From the example, it can be seen that HFSSs could vividly and effectively represent various preferences of different decision-makers and avoid overlooking any subjective intentions of decision-makers. In the future work, HFSSs can be used to deal with much more uncertain problems.

## Figures and Tables

**Table 1:** The tabular representation of the SS (*F*, *E*).

*U*	Beautiful	Cheap	In the green surroundings
*h*_1_	0	1	0
*h*_2_	1	0	0
*h*_3_	0	1	1
*h*_4_	1	0	1

**Table 2:** The tabular representation of the FSS $\left(\tilde{F},E\right)$.

*U*	Beautiful	Cheap	In the green surroundings
*h*_1_	0.2	0.8	0.1
*h*_2_	0.7	0.3	0.2
*h*_3_	0.1	0.7	0.9
*h*_4_	0.7	0.1	0.8

**Table 3:** The tabular representation of the HFSS $\left(\tilde{F},E\right)$.

*U*	Beautiful	Cheap	In the green surroundings
*h*_1_	{0.3,0.2}	{0.9,0.8}	{0.1}
*h*_2_	{0.8,0.7}	{0.3}	{0.3,0.2}
*h*_3_	{0.2,0.1}	{0.7,0.6}	{0.9,0.7}
*h*_4_	{0.7}	{0.1}	{0.8}

**Table 4:** Mr. X's evaluation for the alternative houses.

*U*	Beautiful	Cheap	In the green surroundings
*h*_1_	{0.3,0.2}	{0.9,0.8}	{0.1}
*h*_2_	{0.8,0.7}	{0.3}	{0.3,0.2}
*h*_3_	{0.2,0.1}	{0.7,0.6}	{0.9,0.7}
*h*_4_	{0.7}	{0.1}	{0.8}

**Table 5:** Mrs. X's evaluation for the alternative houses.

*U*	Beautiful	Cheap	In the green surroundings
*h*_1_	{0.8}	{0.8}	{0.9}
*h*_2_	{0.5}	{0.7}	{0.8,0.7}
*h*_3_	{0.7}	{0.4}	{0.3}
*h*_4_	{0.7}	{0.9}	{0.5}

**Table 6:** The evaluation of Mr. X for the alternative houses.

*U*	Beautiful	Cheap	In the green surroundings
*h*_1_	{0.7}	{0.5}	{0.8,0.7}
*h*_2_	{0.8}	{0.8}	{0.5}
*h*_3_	{0.7}	{0.1}	{0.6}
*h*_4_	{0.5,0.4}	{0.9}	{0.7}

**Table 7:** Ranking of the alternatives by utilizing *k*(*t*) = −log⁡(*t*).

	*s*(*μ*_1_)	*s*(*μ*_2_)	*s*(*μ*_3_)	*s*(*μ*_4_)	Ranking
HFSWA	0.683	0.597	0.583	0.695	*s*(*h*_4_) > *s*(*h*_1_) > *s*(*h*_2_) > *s*(*h*_3_)
HFSWG	0.434	0.489	0.422	0.522	*s*(*h*_4_) > *s*(*h*_2_) > *s*(*h*_1_) > *s*(*h*_3_)

**Table 8:** Ranking of the alternatives by utilizing *k*(*t*) = log⁡((2 − *t*)/*t*).

	*s*(*μ*_1_)	*s*(*μ*_2_)	*s*(*μ*_3_)	*s*(*μ*_4_)	Ranking
HFSWA	0.658	0.582	0.563	0.680	*s*(*h*_4_) > *s*(*h*_1_) > *s*(*h*_2_) > *s*(*h*_3_)
HFSWG	0.474	0.506	0.447	0.555	*s*(*h*_4_) > *s*(*h*_2_) > *s*(*h*_1_) > *s*(*h*_3_)

**Table 9:** Ranking of the alternatives by utilizing different *λ* in GHFSWA.

*λ*	*s*(*h*_1_)	*s*(*h*_2_)	*s*(*h*_3_)	*s*(*h*_4_)	The final ranking
*λ* = 1	0.683	0.597	0.583	0.695	*s*(*h*_4_) > *s*(*h*_1_) > *s*(*h*_2_) > *s*(*h*_3_)
*λ* = 2	0.710	0.617	0.612	0.716	*s*(*h*_4_) > *s*(*h*_1_) > *s*(*h*_2_) > *s*(*h*_3_)
*λ* = 5	0.756	0.664	0.662	0.752	*s*(*h*_1_) > *s*(*h*_4_) > *s*(*h*_2_) > *s*(*h*_3_)
*λ* = 10	0.788	0.703	0.698	0.783	*s*(*h*_1_) > *s*(*h*_4_) > *s*(*h*_2_) > *s*(*h*_3_)

**Table 10:** Ranking of the alternatives by utilizing different *λ* in GHFSWG.

*λ*	*s*(*h*_1_)	*s*(*h*_2_)	*s*(*h*_3_)	*s*(*h*_4_)	The final ranking
*λ* = 1	0.434	0.489	0.422	0.522	*s*(*h*_4_) > *s*(*h*_2_) > *s*(*h*_1_) > *s*(*h*_3_)
*λ* = 2	0.409	0.470	0.385	0.462	*s*(*h*_2_) > *s*(*h*_4_) > *s*(*h*_1_) > *s*(*h*_3_)
*λ* = 5	0.366	0.437	0.309	0.378	*s*(*h*_2_) > *s*(*h*_4_) > *s*(*h*_1_) > *s*(*h*_3_)
*λ* = 10	0.334	0.399	0.255	0.318	*s*(*h*_2_) > *s*(*h*_1_) > *s*(*h*_4_) > *s*(*h*_3_)

## References

[1] Zadeh L. A. (1965). Fuzzy sets. *Information and Computation*.

[2] Bellman R. E., Zadeh L. A. (1970). Decision-making in a fuzzy environment. *Management Science*.

[3] Yager R. R. (1977). Multiple objective decision-making using fuzzy sets. *International Journal of Man-Machine Studies*.

[4] Zadeh L. A. (1975). Fuzzy logic and approximate reasoning. *Synthese*.

[5] Pedrycz W. (1990). Fuzzy sets in pattern recognition: methodology and methods. *Pattern Recognition*.

[6] Atanassov K. T. (1986). Intuitionistic fuzzy sets. *Fuzzy Sets and Systems*.

[7] Atanassov K. T. (1999). *Intuitionistic Fuzzy Sets*.

[8] Atanassov K. T., Gargov G. (1989). Interval valued intuitionistic fuzzy sets. *Fuzzy Sets and Systems*.

[9] Shu M.-H., Cheng C.-H., Chang J.-R. (2006). Using intuitionistic fuzzy sets for fault-tree analysis on printed circuit board assembly. *Microelectronics Reliability*.

[10] Wang J.-Q., Nie R., Zhang H.-Y., Chen X.-H. (2013). New operators on triangular intuitionistic fuzzy numbers and their applications in system fault analysis. *Information Sciences*.

[11] Liu P.-D., Liu Y. (2014). An approach to multiple attribute group decision making based on intuitionistic trapezoidal fuzzy power generalized aggregation operator. *International Journal of Computational Intelligence Systems*.

[12] Wang J.-Q., Nie R.-R., Zhang H.-Y., Chen X.-H. (2013). Intuitionistic fuzzy multi-criteria decision-making method based on evidential reasoning. *Applied Soft Computing Journal*.

[13] Wang J.-Q., Zhang H.-Y. (2013). Multicriteria decision-making approach based on Atanassov's intuitionistic fuzzy sets with incomplete certain information on weights. *IEEE Transactions on Fuzzy Systems*.

[14] Wang J.-Q., Han Z.-Q., Zhang H.-Y. (2014). Multi-criteria group decision-making method based on intuitionistic interval fuzzy information. *Group Decision and Negotiation*.

[15] Torra V. (2010). Hesitant fuzzy sets. *International Journal of Intelligent Systems*.

[16] Torra V., Narukawa Y. On hesitant fuzzy sets and decision.

[17] Xia M., Xu Z. (2011). Hesitant fuzzy information aggregation in decision making. *International Journal of Approximate Reasoning*.

[18] Zhu B., Xu Z. S., Xia M. (2012). Hesitant fuzzy geometric Bonferroni means. *Information Sciences*.

[19] Wei G. (2012). Hesitant fuzzy prioritized operators and their application to multiple attribute decision making. *Knowledge-Based Systems*.

[20] Xia M., Xu Z., Chen N. (2013). Some Hesitant fuzzy aggregation operators with their application in group decision making. *Group Decision and Negotiation*.

[21] Wang J.-Q., Wang D.-D., Zhang H. Y., Chen X. H. (2013). Multi-criteria outranking approach with hestitant fuzzy sets. *OR Spectrum*.

[22] Yu D., Wu Y., Zhou W. (2011). Multi-criteria decision making based on Choquet integral under hesitant fuzzy environment. *Journal of Computational Information Systems*.

[23] Chen N., Xu Z. S., Xia M. (2013). Correlation coefficients of hesitant fuzzy sets and their applications to clustering analysis. *Applied Mathematical Modelling*.

[24] Xu Z. S., Xia M. M. (2011). Distance and similarity measures for hesitant fuzzy sets. *Information Sciences*.

[25] Xu Z., Xia M. (2011). On distance and correlation measures of hesitant fuzzy information. *International Journal of Intelligent Systems*.

[26] Molodtsov D. (1999). Soft set theory—first results. *Computers & Mathematics with Applications*.

[27] Maji P. K., Biswas R., Roy A. R. (2003). Soft set theory. *Computers & Mathematics with Applications*.

[28] Maji P. K., Roy A. R., Biswas R. (2002). An application of soft sets in a decision making problem. *Computers and Mathematics with Applications*.

[29] Ali M. I., Feng F., Liu X., Min W., Shabir M. (2009). On some new operations in soft set theory. *Computers and Mathematics with Applications*.

[30] Sezgin A., Atagün A. O. (2011). On operations of soft sets. *Computers & Mathematics with Applications*.

[31] Jiang Y. C., Tang Y., Chen Q. M., Cao Z. (2011). Semantic operations of multiple soft sets under conflict. *Computers & Mathematics with Applications*.

[32] Min W. K. (2012). Similarity in soft set theory. *Applied Mathematics Letters*.

[33] Aktaş H., Çağman N. (2007). Soft sets and soft groups. *Information Sciences*.

[34] Acar U., Koyuncu F., Tanay B. (2010). Soft sets and soft rings. *Computers & Mathematics with Applications*.

[35] Gong K., Xiao Z., Zhang X. (2010). The bijective soft set with its operations. *Computers and Mathematics with Applications*.

[36] Babitha K. V., Sunil J. J. (2010). Soft set relations and functions. *Computers & Mathematics with Applications*.

[37] Ali M. I., Shabir M., Naz M. (2011). Algebraic structures of soft sets associated with new operations. *Computers & Mathematics with Applications*.

[38] Ali M. I. (2012). Another view on reduction of parameters in soft sets. *Applied Soft Computing Journal*.

[39] Çağman N., Enginoğlu S. (2010). Soft set theory and uni-int decision making. *European Journal of Operational Research*.

[40] Feng F., Li Y., Çağman N. (2012). Generalized *uni-int* decision making schemes based on choice value soft sets. *European Journal of Operational Research*.

[41] Maji P. K., Biswas R., Roy A. R. (2001). Fuzzy soft sets. *Journal of Fuzzy Mathematics*.

[42] Borah M., Neog T. J., Sut D. K. (2012). A study on some operations of fuzzy soft sets. *International Journal of Modern Engineering Research*.

[43] Guan X. C., Li Y., Feng F. (2013). A new order relation on fuzzy soft sets and its application. *Soft Computing*.

[44] Feng F., Fujita H., Jun Y. B., Khan M. (2014). Decomposition of fuzzy soft sets with finite value spaces. *The Scientific World Journal*.

[45] Maji P. K., Biswas R., Roy A. R. (2001). Intuitionistic fuzzy soft sets. *Journal of Fuzzy Mathematics*.

[46] Xu W., Ma J., Wang S., Hao G. (2010). Vague soft sets and their properties. *Computers & Mathematics with Applications*.

[47] Zhou X., Li Q. (2014). Generalized vague soft set and its lattice structures. *Journal of Computational Analysis and Applications*.

[48] Yang X. B., Lin T. Y., Yang J., Li Y., Yu D. (2009). Combination of interval-valued fuzzy set and soft set. *Computers and Mathematics with Applications*.

[49] Ma X., Qin H., Sulaiman N., Herawan T., Abawajy J. H. (2014). The parameter reduction of the interval-valued fuzzy soft sets and its related algorithms. *IEEE Transactions on Fuzzy Systems*.

[50] Jiang Y., Tang Y., Liu H., Chen Z. (2013). Entropy on intuitionistic fuzzy soft sets and on interval-valued fuzzy soft sets. *Information Sciences*.

[51] Majumdar P., Samanta S. K. (2010). Generalised fuzzy soft sets. *Computers & Mathematics with Applications*.

[52] Roy A. R., Maji P. K. (2007). A fuzzy soft set theoretic approach to decision making problems. *Journal of Computational and Applied Mathematics*.

[53] Feng F., Jun Y. B., Liu X., Li L. (2010). An adjustable approach to fuzzy soft set based decision making. *Journal of Computational and Applied Mathematics*.

[54] Kong Z., Wang L., Wu Z. (2011). Application of fuzzy soft set in decision making problems based on grey theory. *Journal of Computational and Applied Mathematics*.

[55] Mitra Basu T., Mahapatra N. K., Mondal S. K. (2012). A balanced solution of a fuzzy soft set based decision making problem in medical science. *Applied Soft Computing Journal*.

[56] Xiao Z., Chen W., Li L. (2012). An integrated FCM and fuzzy soft set for supplier selection problem based on risk evaluation. *Applied Mathematical Modelling: Simulation and Computation for Engineering and Environmental Systems*.

[57] Li Z. W., Wen G. Q., Han Y. (2014). Decision making based on intuitionistic fuzzy soft sets and its algorithm. *Journal of Computational Analysis and Applications*.

[58] Çağman N., Karataş S. (2013). Intuitionistic fuzzy soft set theory and its decision making. *Journal of Intelligent & Fuzzy Systems*.

[59] Jiang Y., Tang Y., Chen Q. (2011). An adjustable approach to intuitionistic fuzzy soft sets based decision making. *Applied Mathematical Modelling: Simulation and Computation for Engineering and Environmental Systems*.

[60] Xiao Z., Yang X., Niu Q. (2012). A new evaluation method based on D-S generalized fuzzy soft sets and its application in medical diagnosis problem. *Applied Mathematical Modelling*.

[61] Xiao Z., Xia S., Gong K., Li D. (2012). The trapezoidal fuzzy soft set and its application in MCDM. *Applied Mathematical Modelling*.

[62] Zhang H., Shu L., Liao S. (2014). Generalized trapezoidal fuzzy soft set and its application in medical diagnosis. *Journal of Applied Mathematics*.

[63] Zhang Z. (2012). A rough set approach to intuitionistic fuzzy soft set based decision making. *Applied Mathematical Modelling*.

[64] Babitha K. V., John S. J. (2013). Hesitant fuzzy soft sets. *Journal of New Results in Science*.

[65] Klir G. J., Yuan B. (1995). *Fuzzy Sets and Fuzzy Logic: Theory and Applications*.

[66] Nguyen H. T., Walker R. A. (1997). *A First Course in Fuzzy Logic*.

[67] Klement E. P., Mesiar R. (2005). *Logical, Algebraic, Analytic, and Probabilistic Aspects of Triangular Norms*.

[68] Beliakov G., Pradera A., Calvo T. (2007). *Aggregation Functions: A Guide for Practitioners*.

[69] Chen N., Xu Z., Xia M. (2013). The electre I multi-critria decision-making method based on hesitant fuzzy sets. *International Journal of Information Technology and Decision Making*.

